# Stimulating effect of biogenic nanoparticles on the germination of basil (*Ocimum basilicum* L.) seeds

**DOI:** 10.1038/s41598-023-50654-8

**Published:** 2024-01-19

**Authors:** Aziz Sencan, Semra Kilic, Havva Kaya

**Affiliations:** 1 Department of Chemical Engineering, Suleyman Demirel University, 32260 Isparta, Turkey; 2 Department of Biology, Suleyman Demirel University, 32260 Isparta, Turkey; 3 Department of Bioengineering, Suleyman Demirel University, 32260 Isparta, Turkey

**Keywords:** Plant embryogenesis, Plant morphogenesis, Biochemistry, Chemical biology

## Abstract

Metal nanoparticles synthesized using various biosources are the subject of focus in many research areas thanks to their improved biological effects and increased bioavailability. Silver (Ag), zinc oxide (ZnO) and magnetite (Fe_3_O_4_) nanoparticles (NPs) were obtained by using low-cost, low-energy, environmentally friendly, non-toxic chemicals and easily accessible thyme leaves and lavender flowers. The effects of various concentrations of biosynthesized NPs on the germination and germination index of basil seeds were defined comparatively. Phytochemicals in lavender flower extract acted as reducing and capping agents in the biosynthesis of Ag-NPs, and phytochemicals in thyme leaves extract acted for the biosynthesis of ZnO-NPs ve Fe_3_O_4_-NPs. Relative root length was detected at 25 mg/L ZnO-NP, stem length at 50 mg/L ZnO-NP, and relative seed germination 100 mg/L Fe_3_O_4_-NP with the maximum value. However, germination percentage, germination index, germination vigor index and root length were found to be maximum compared to other NP applications at Ag-NPs at 200 mg/L. This research showed that the germination promoting effects of NPs, which may be essential microelements, are related to their size, surface area, morphology and concentration. Thus, it promoted early and rapid germination by breaking the NP's seed dormancy.

## Introduction

The potential agricultural use of nanotechnology, which is becoming increasingly widespread in various fields of industry such as health, medicine, cosmetics, food and feed, environmental health, mechanics, optics, biomedical sciences, chemical industries, electronics, space industries, energy science, are important scientific developments that will be hand down the next generations^1,2^. The most feasible and simple way to method improve seed quality and increase crop yield is the administer metal nanoparticles (NPs) to seeds before sowing^3^. For this purpose, NPs are produced by physical and chemical processes that can be controlled, have various morphologies and chemical compositions, and are very small (1–100 nm) and much more effective. However, these methods cause both pollution and high costs, since they require toxic chemicals and high energy consumption^4^. Due to extraordinary drought, precipitation and temperature changes occurring in our world where toxic materials are gradually increasing disturb the ecosystem balance, it has become a necessity for products such as fertilizers, herbicides, pesticides, fungicides used in the food and agriculture industry to have the least toxic effect possible. The use of ecologically friendly phyto- NPs, which can meet future agricultural demands, improve product quality and yield, and reduce chemical pollution, is to matter for this reason. Due to their high surface-to-volume ratio, high reactivity, dissolution properties, and strength, as well as their catalytic, magnetic, electrical, mechanical, optical, chemical, and biological properties, NPs have become a focus in sustainable agriculture over the past 20 years^5^. However, the green synthesis approach, which uses biological extracts as chemical reducing agents, begun to replace chemical synthesis, which is unfavorable due to much higher energy consumption and negative environmental effects in the production of NPs^6^. Due to its low cost, minimal chemical use, environmental friendliness, and high performance capabilities, green synthesis is more preferred. For instance, it has been observed that NPs formed through green synthesis connect with biomolecules including protein, enzyme, and sugar readily and exhibit more activity than NPs obtained through chemical or physical approaches^7^. Biological materials have biomolecules that reduce metal ions to NPs of desired shape and size, thanks to the limiting, reducing and stabilizing, phytochemicals (such as polyphenols, flavonoids, terpenoids, alkaloids, tannins, and alcoholic compounds) and biomolecules(such as polysaccharides, amino acids, organic acids, and vitamins) necessary to inhibit the aggregation/agglomeration process. These capping agents enable biosynthesized NPs to disperse superbly in colloidal solution^8^. Additionally, the biological extract concentration, pH, temperature, salt concentration, and exposure period all affect the size and shape of NPs^9^.

Basil (*Ocimum basilicum* L.) has a wide range of uses in traditional medicine, in the treatment of various diseases such as carminative, cough, diarrhea, rheumatism, constipation, cancer, and in the field of cosmetics, due to having bioactive essential oils such as α-Pinene, β-myrcene, eucalyptol, cis-linaloloxide, citral, eugenol, copaene, humulene, nerolidol. In recent studies, it has been found that the active substances isolated from basil are highly effective in various bacteria, but the effectiveness of basil is less than commercial antibiotics^10^. Therefore, improving the quantity and quality of basil's secondary metabolite content will increase of therapeutic effect. It has become crucial to grow these plants, improve their cultivation and growth mechanisms on industrial platforms, and increase the quality and amount of the secondary metabolites they contain to meet the increasing demand for these plants, which have a variety of uses in the pharmaceutic, cosmetic, and food industries. According the recent studies, biogenic obtained NPs overcome nutrient deficiencies in seed germination and seedling growth, act as agents that promote cell division and elongation^11^, promote growth by causing changes in gene, regulate enzymatic processes in various biochemical reactions, cytokinin and gibberellin during germination, accelerate this process by affecting the activity of the plant, activating the bacteria that encourage plant growth, and making it easier for them to attach to the plant roots^12^. In addition, the use of phytoparticles makes plants more resistant to both biotic and abiotic stressors, ensuring that plants are minimally affected by these stress factors during the germination and growth processes. When metal ions are exposed to the phytochemicals of the plant extract, they are reduced to elemental metals and then to NPs, and their optical properties change^13^, so they activate or accelerate some protective mechanisms, promoting plant growth and development. For their safe usage, it is essential to consider the potential effects of NPs on seed germination and seedling growth, which are intended to be employed to satisfy the rising global demand and increase plant production in sustainable agriculture methods. For this purpose, Ag-NP, ZnO-NP and Fe_3_O_4_-NPs were biosynthesized by using aqueous extracts of lavender flowers (Ag) and thyme leaves (Zn and Fe) containing secondary metabolites. Different concentrations of NP were optimized for maximum yield in germination of basil seeds, their potential effects in this process were identified by comparing them.

## Experimental details

### Preparation of plant materials and extracts

Nanoparticles (NPs) were synthesized using lavender (*Lavandula officinalis* L.) flowers and thyme leaves (*Origanum minutiflorum* O. Schwarz & P.H. Davis). The thyme (Sutçuler/Isparta) plant samples were collected by random sampling from the areas where they are distributed in Isparta and its surroundings in June 2022. The species identification according to the Flora of Turkey^14^ was made by Semra Kilic at Süleyman Demirel University, Department of Botany. The plant samples were archived as herbarium material with the codes Kilic-5211/2022. Lavander leaves ve basil seeds were purchased from a herbalist selling commercial products. Experimental research, field studies on plants and the collection of plant material, comply with relevant institutional, national, and international guidelines and legislation. The plant parts were dried in the shade at room temperature for two days. We pulverized the dry plant samples in the blender. 1 g. plant samples were infused in 100 ml of boiled water for 20 min and Whatman.

### Biosynthesis of NPs

Silver NPs (Ag-NPs) were obtained by adding 50 ml of lavender extract to 50 ml of 1 mM silver nitrate (AgNO3: 0.017 mg in 100 ml of Milli-Q water) solution. The mixture, which turned blackish/dark brown, was centrifuged at 600 rpm for 15 min.

Zinc oxide NPs (ZnO-NPs) were prepared by mixing 0.1 M zinc chloride (ZnCl_2_.4H_2_O) in 100 ml of distilled water at 600 rpm. 50 mL of this mixture was taken and 50 mL of thyme extract was added. After the solution was stirred with a magnetic stirrer (200 rpm) at 25 °C for 24 h, a white precipitate was obtained.

To obtain iron oxide NPs (magnetite: Fe_3_O_4_-NPs), 0.01 molar solutions of ferric chloride tetrahydrate (FeCl_2_.4H_2_O) and ferric chloride hexahydrate (FeCl_3_.6H_2_O) were prepared. 50 mL of this mixture was taken and 50 mL of thyme extract was added. Waited until the color of the mixture turned reddish/brown. The mixture was centrifuged at 6000 rpm for 30 min. All NPs obtained by phyto-nanosynthesis were dried in a vacuum oven, weighed, and stored at + 4 °C in a dark capped vial for germination experiments.

### Biological applications of phytosynthesized NPs

In order to determine the effect of several concentrations of Ag-NPs, ZnO-NPs, and Fe_3_O_4_-NPs on the germination parameters of basil (*Ocimum basilicum* L.) seeds, Ag-NPs 0 (control), 50, 100, 200, 400 mg/L; ZnO-NPs 0 (control), 25, 50, 100, 200 mg/L; Fe_3_O_4_-NPs and 0 (control), 25, 50, 100, 200 mg/L, concentrations were applied to basil seeds. Approximately 60 to basil seed were primed for 24 h at each NPs concentration. For each experiment, 20 seeds of the same size were carefully selected and placed on Whatman papers soaked with 20 ml of distilled water in petri dishes, and should be incubated at 25 °C ± 2 for 7 days. Each experiment was repeated 3 times. The seeds started to germinate on the 3rd day. The ledge of radicle through the seed coat was taken as the criteria of seed germination. The following parameters were used to define the *Germination Index* (*GI*)^15^ and *Germination Vigor Index* (*VI*)^16^.

The germination index (*GI*) is the measure of relative seed germination and relative root elongation; the germination vigor index (*GVI*) is the measure of seedlings length [(root length + shoot length) (mm) and germination percentage (%)] were calculated according to Equations;

$${\text{Relative seed germination \% }} = \frac{{\text{Seeds germination in treatment }}}{{\text{Seeds germination in control}}} \times 100$$

$${\text{Relative root growth \% }} = \frac{{\text{Mean root length in treatment }}}{{\text{Mean root length in control}}} \times { }100$$

$${\text{GI}} = \frac{{\left( {{\text{\% Relative seed germination}}} \right){ } \times { }\left( {{\text{\% Relative root growth}}} \right){ }}}{100}$$

$${\text{GVI}} = \frac{{{\text{Seedlings length }}\left( {{\text{mm}}} \right)\left( {{\text{root length}} + {\text{shoot length}}} \right){ } \times {\text{ Germination percentage }}\left( {\text{\% }} \right){ }}}{100}$$

The following formula was used to calculate the *seed water content* (*SWC*), weight of fresh germinated seeds at the end of germination (*FW*), and weight of the same seeds after drying at 70 °C (*DW*) (0.0001 g).

$${\text{SWC}} = \frac{{{\text{FW }} \times {\text{DW }}}}{{{\text{FW}}}} \times { }100{ }$$

### Statistical analysis

Trilicated data of each experiment was analyzed statistically using SPSS software, version 21 (IBM SPSS), and the obtained values were expressed as mean values ± standard deviation (SD). The experimental data were analysed by using one-way analysis (ANOVA), and the mean values for each treatment were compared using the Duncan’s multiple range test at the P ≤ 0.05 confidence level.

## Results and discussion

NPs are widely used in agriculture for their various benefits, including their usability, biocompatibility, and biodegradability. In order to improve yield and quality, reduce on nutrient losses, and control plant diseases, some metal-based NPs including also Ag-NPs, ZnO-NPs, Fe_3_O_4_-NPs are increasingly used as a fertilizer source. Since the small size of NPs facilitates their passage through the cell plasma membrane, this has significant advantages such as increasing the amount and activity in the target structure.

The positive effects of biogenic NPs on seed germination can also be attributed to their small size, higher surface-to-volume ratio, and synergistic effects from physicochemical interactions with phytochemicals that formed in a coating on crystalline NPs with great optical qualities^17^. Because of this, using NPs produced by green synthesis which is low-cost, accessible, and ecologically friendly is preferred to NPs made by chemical synthesis. NPs support to the healthy and rapid progress of the germination processes of seeds by making important contributions to the metabolic functioning of most macromolecules such as protein, fat, carbohydrates and vitamins. For this purpose, the effects of different concentrations of biogenic produced NPs on the germination of basil seeds; Germination percentage (%), root and stem lengths (mm), fresh and dry weights (g) were taken into account, and Germination Index (*GI*), Germination Vigor Index (*GVI*) and Seed Water Content (*SWC*) results were evaluated.

### The effect of Ag-NPs

**Figure 1:**
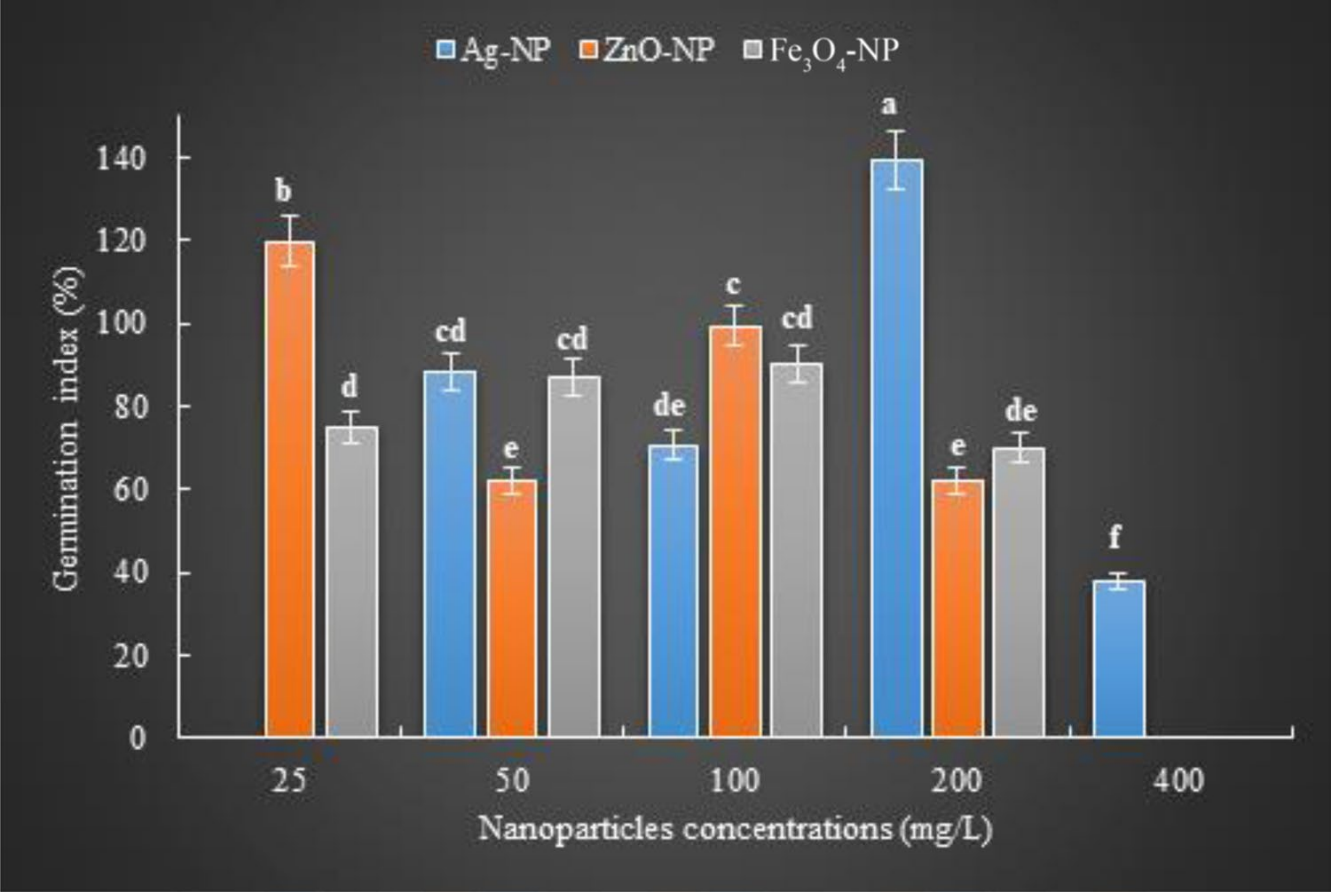
Response of NPs in germination index. Bars indicate standard errors of the means ± SE (*n* = 20); different letters over identical bars indicate significant differences (Duncan post-hoc test; *P* ≤ 0.05).

**Figure 2:**
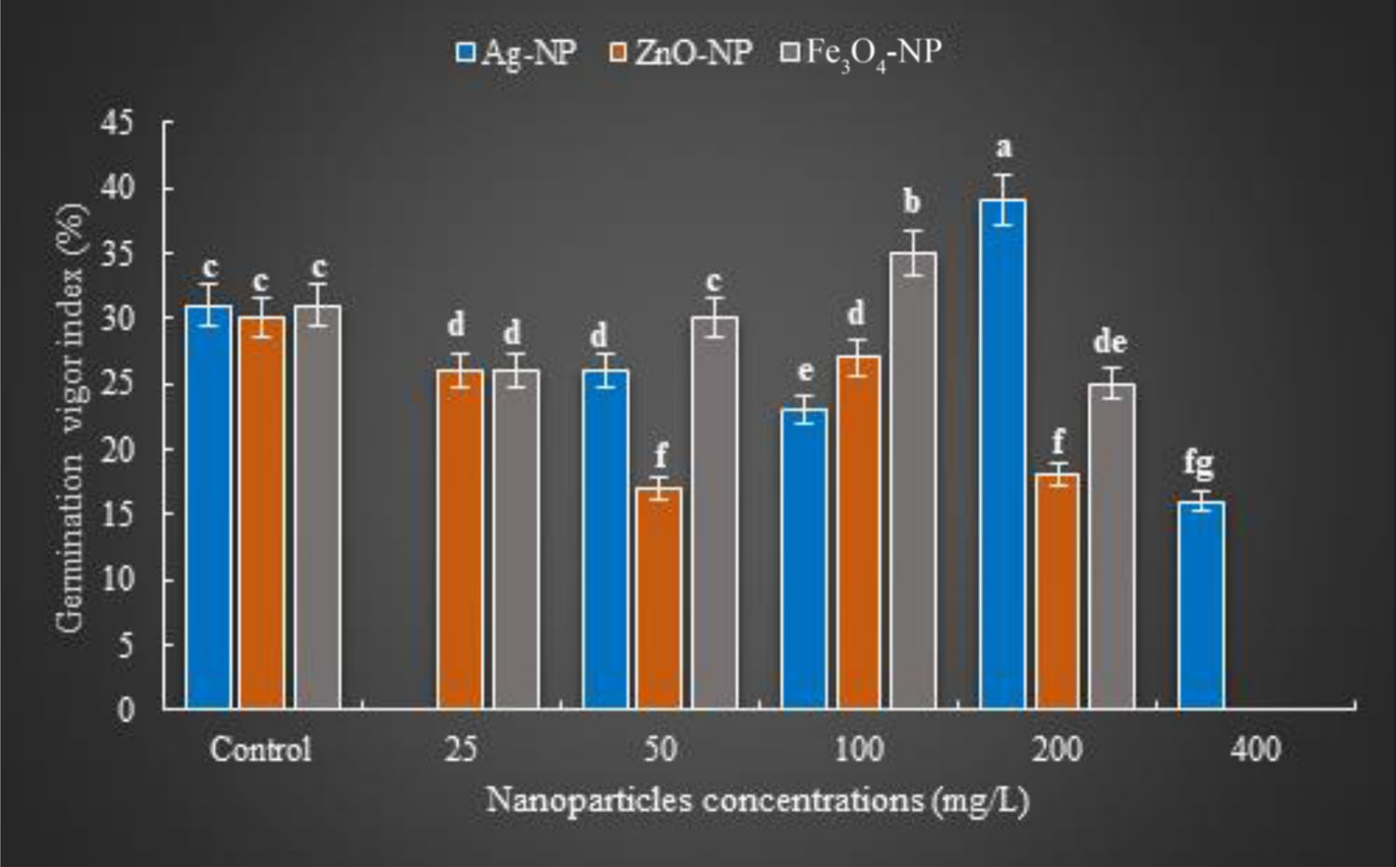
Response of NPs germination vigor index. Bars indicate standard errors of the means ± SE (*n* = 20); different letters over identical bars indicate significant differences (Duncan post-hoc test; *P* ≤ 0.05).

The effect of different concentrations of Ag-NPs on the germination of basil seeds was described by germination index (*GI*) data, which was revealed by evaluating the results of relative seed germination and relative root growth. The effect of Ag-NPs on the germination percentage and root length of basil seeds changed by its concentration (P ≤ 0.05). Accordingly, the germination percentage is 70%, 65%, 60%, 85% and 45%; root lengths of 16.8, 16.4, 13.8, 19.4 and 10.4 mm, at control, 50, 100, 200 and 400 mg/L concentrations, respectively. Significant increases in both germination percentage and root length were observed at 200 mg/L concentration of Ag-NPs. At the concentration of 200 mg/L, these data demonstrated a germination index of 139.15%, reflecting relative seed germination of 121% and relative root growth of 115%. While this value increased rapidly at its 50 and 100 mg/L concentrations, it resulted in a sharp decrease at 400 mg/L (Fig. 1.). This indicates that low concentrations of Ag-NPs promote germination, while high concentrations inhibit it. Ag-NPs contribute to germination by promoting water and mineral uptake, due to effective nutrient absorption in the seed coat and its ability to form new pores that promote germination. The differences in biometric and biochemical parameters of three vegetable species, expanded the scope of knowledge on their micropropagation, especially at 100 mg/L concentration, Ag-NPs increase the permeability of the cell membrane and provide better water uptake^18^. Germinatin vigor index (*GVI*) obtained by considering seedling size (root + stem length mm) and germination percentage data decreased by 17%, 26% ve 49% at 50, 100 ve 400 mg/L, respectively, and increased by 21% at 200 mg/L, compared to control (P ≤ 0.05). The total length of the roots and stems was maximum (44.6 mm), at 200 mg/L Ag-NP concentration (Fig. 2.). Additionally, the germination percentage was the highest at 115% at the similar concentration (compared to other treatments, including control) resulting in the highest *GVI* at this concentration. Interestingly, Ag-NPs administration had a beneficial effect at 200 mg/L despite negatively affecting *GVI* with in risen concentration. The fact that this result is reversed at 400 mg/L and even lower than other applications (50 and 100 mg/L) may be due to the toxic effect that excessively accumulated Ag^+^ in the tissues. Ag-NPs increase *GVI* at appropriate concentrations by stimulating various metabolic mechanisms (such as α-amylase activity, reactive oxygen species (ROS) production, and antioxidant systems) associated with germination by increasing water and small solute uptake and movement, starch hydrolysis and seed reserve mobilization during seed germination. Priming applied Ag-NPs, stimulates seed germination by breaking seed dormancy by affecting various biochemical processes such as hydrolysis and growth-suppressing metabolites, absorption, and enzyme activation^19^. On the other hand, the fact that NPs increase the mitotic index by affecting the cell division stages may explain the variability in root and stem lengths that are still in the growth phase. Since the application of appropriate concentrations of Ag-NPs due to the increases seed viability, thanks to inducing reactive oxygen species (ROS) necessary for early growth and development of embryos, as well as increasing synchronization of germination by retorting metabolic enzymes^20^, AgNP administration positively affected *GVI* at 200 mg/L, while lower and higher concentrations negatively affected it.

The seed water content (*SWC*), which was calculated using the results of the weight of the seeds (*FW*) and the weight of the same seeds after drying at 70 °C (*DW*) data at the end of germination by various concentrations of Ag-NPs, was also used to determine the effect of the AgNP on the germination of basil seeds. *SWC* increased by 29%, 12%, 31% and 37% compared to control at 50, 100, 200 and 400 mg/L, respectively. Interestingly the rate of increase of *SWC* at 100 mg/L decreases and this value increases with rising concentration. This effect resulted in basil seeds being positive at 50 mg/L and decreasing at 100 mg/L, but further increasing again (compared to 50 mg/L) at 200 and 400 mg/L. The fact that the *FW*/*DW* value, at which the estimated cell water content can be determined, changes with the NP concentration indicate Ag-NPs affect the germination process by maintaining sufficient water intake for germination and cell turgor. Ag-NPs promote seed germination and growth since they increase nutrient uptake and transport at the proper concentration^21^.

### The effect of ZnO-NPs

The effect of different concentrations of ZnO-NPs and their effects on the germination index generated by the relative seed germination and relative root growth data caused the *GI* to change gradually at different concentrations of ZnO-NPs (Fig. 1.). However, the change of *GI* occurred by root length differences (P ≤ 0.05), and the difference in germination percentage was insignificant at the concentration change (P ≥ 0.05). Germination percentage decreased by 15%, 43%, 8%, and 29% at 25, 50, 100, and 200 mg/L concentration, respectively, compared to the control. Root length increased by 27%, 9%, 8% at 25, 50 and 100 mg/L, respectively, and decreased by 15% at 200 mg/L, compared to control. The maximum relative root length was 136% at 25 mg/L (16.6 mm), while the maximum relative seed germination was determined as 92% at 100 mg/L. Therefore, the *GI* was highest at 25 mg/L with 119.68%. ZnO-NPs improve seed germination, seedling emergence, and growth, as they play an important role in the biosynthesis of endogenous hormones such as auxin and gibberellin, and act as an important component in carbohydrate and protein metabolism, breaking dormancy to initiate the germination process^22^. However, due to the seed coat penetration properties of NPs, ZnO-NP positively affected germination at low concentration (25 mg/L), while it caused toxic effects at higher concentrations. The fact that *GI* parallels the increase in ZnO-NP (25 mg/L) concentration can be explained by the need for Zn, which is the most critical micronutrient, for the activation of enzymes involved in protein and carbohydrate synthesis, nucleic acid and lipid regulation, and the breaking of primary seed dormancy^23^. Higher concentrations negatively affected the *GI* due to damage to plant cells, even causing toxic effects. Since the high concentration of ZnO-NPs, which has a high photocatalytic effect, promotes ROS production, excessive ROS accumulation causes membrane lipid peroxidation and resulting in cell death^24^. Therefore determining the optimal concentrations for administering ZnO-NP is crucial. The linear association between the rise in Zn concentration and protein synthesis accounts for the high Zn content in the root tips and coleoptile, therefore Zn is highly active during the germination phase of seeds^25^. The difference between concentrations was insignificant in the germination vigor index (*GVI*) (P ≥ 0.05) (Fig. 2.). The highest root length was measured at 25 mg/L (16.6 mm), and the highest stem length was measured at 50 mg/L (30.6 mm), however, the maximum germination percentage (65%) was found at 100 mg/L, so that *GVI* obtained the highest value (27%) at 100 mg/L. The detection of maximum values in root and stem lengths at different concentrations of ZnO-NPs showed that the root closest to the embryonic axis first benefited from the positive effect of Zn, but the stem far from the embryonic axis perceived the positive effect of higher concentrations of Zn. Because after entering the water-soluble Zn micropyle and hilum, it is distributed from the embryonic axis to the cotyledons, where the first root exit organized. Since Zn uptake and distribution from cotyledons during seed germination was not associated with seed viability^26^, the similarity of this result with control application (26%) showed that ZnO-NPs concentrations were not effective on *GVI*. According to the *GVI* results, defined as the coordinated and sequential sustained activity of the metabolic activity of the plant embryo, ZnO-NPs did not show discernible effects in this process. The effectivity and functionality of ZnO-NPs may vary depending on plant variety and age as well as the applied concentration. For instance, when maize seeds, a monocot plant, were administered with various concentrations of ZnO-NPs, the germination percentage was recorded at 100 mg/L with 87% on the eighth day of germination. Shoot length (12.1 cm), root length (20 cm), root and shoot width (1 and 2.8 mm) were detected (at the same concentration), compared to control^27^.

**Figure 3:**
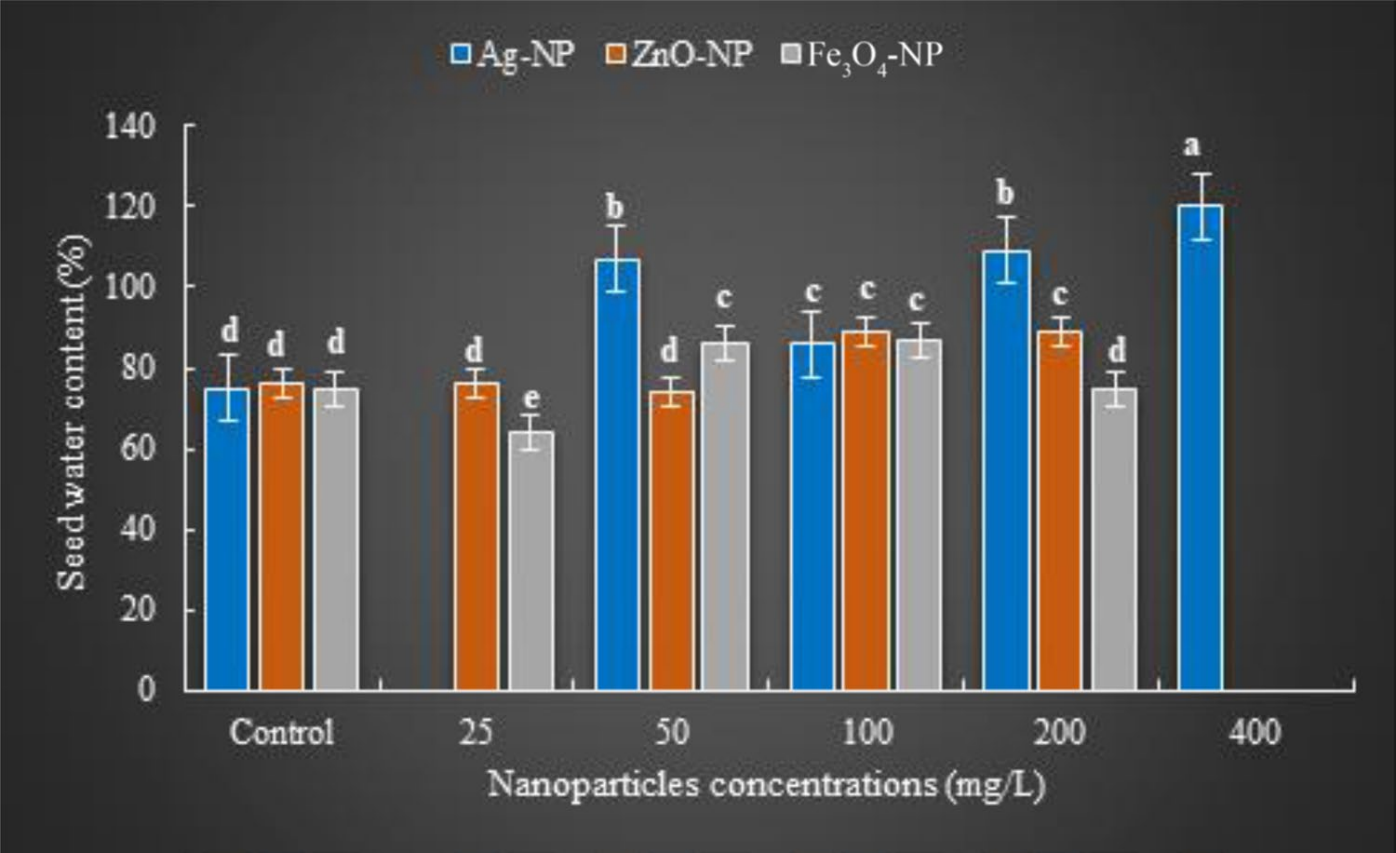
Effects of NPs in seed water content. Bars indicate standard errors of the means ± SE (*n* = 20); different letters over identical bars indicate significant differences (Duncan post-hoc test; *P* ≤ 0.05).

As the ZnO concentrations increased, the water seed content also changed. Although Zn is toxic, it is the basic component of thousands of proteins in plants and is a micronutrient that is active from germination to all other vital processes of the plant's life cycle, as it is part of the transcription factor family known as "zinc fingers" that control the proliferation and differentiation of cells^28^. The effect of different concentrations of ZnO-NPs on the seed water content of basil seeds was calculated by the fresh and dry weights obtained after 20 days of germination (Fig. 3.). Fresh and dry weights of seeds were 0.201 g and 0.009 g, 100 mg/L, respectively. The amount of *SWC* increased with rising concentration of ZnO-NPs, compared to control. This parameter, in which the amount of substance is defined, indicates the increase in the amount of Zn penetrating the seeds with the increase in ZnO-NP concentration. The *SWC* values increase with ZnO-NP concentration, as the increase in the amount of Zn entry from the seed coat and the rate of entry will positively affect its nitrogen metabolism during germination and increase protein production. Since ZnO NPs administration provide a significant increase in dry weight, relative water uptake of seeds, and seedling root length with the presence of appropriate zinc level in the seed, the germination process is positively affected^29^. ZnO-NPs promoted *GI* and *GVI* at certain concentration, showed toxic effect at higher concentrations (50 mg/L and above), causing a decrease in these data. However, it was quite interesting that the positive effects, which are that the *SWC* value increases as the rise of ZnO-NP concentrations, and that high concentrations of Zn did not have a toxic effect, continue depending on the rise in concentration. The phytotoxic effect of zinc, which is a second transition metal in organisms, can be alleviated or even eliminated by administering this metal to plants in NP size.

### The effect of Fe_3_O_4_-NPs

The effect of different concentrations of Fe_3_O_4_-NPs the maximum value of the germination index obtained using data on relative seed germination and relative root growth, was 90% at a concentration of 100 mg/L Fe_3_O_4_-NPs (P ≤ 0.05). This value was provided by the increase of germination percentage. Because, while the germination percentage was 88% at 100 mg/L, the root length at the same concentration was 13.2 mm, which was the lowest of all treatments (Fig. 1.). In other words, while there was a clear correlation between the concentration rise of Fe_3_O_4_-NPs and the increase in germination percentage (this effect was reversed at 200 mg/L), it did not have a corresponding effect on root length. For instance, the germination percentage and root length were 70%, 61%, 73%, 88%, and 61% and measured 18.2, 15.8, 15.4, 13.2, and 14.6 mm, at concentrations of 25, 50, 100, and 200 mg/L Fe_3_O_4_-NPs, respectively. These results showed that with the increase in the concentration of Fe_3_O_4_-NPs showing peroxidase-like activity and the deterioration of the seed pericarp was promoted^30^, and an increase in the germination percentage was recorded. This explains the reason why *GI*, which is known to be directly proportional to germination, increases without being affected by these results, although root lengths decrease with increasing concentration. At the same time, Fe, which is bound to the Phytoferritin protein, is released with phytoferritin decomposition during seed germination, and triggers the seed germination process by supporting the formation of hydroxyl ions that break down the protein layer of the seed^31^. On the other hand, the change of germination percentages with NP concentrations can be explained by the positive effect of Fe_3_O_4_-NPs on cell membrane permeability, increasing water and nutrient uptake and translocation^32^.

Fe_3_O_4_-NPs concentration differences caused significant changes in *GVI* (Fig. 2.). The *GVI*, which was 31% in the control group, was found to be 26% and 31% at 25 and 50 mg/L, respectively, showed a reduction. However, this ratio was the maximum (35%) for all applications at 100 mg/L. *GVI* was negatively affected at a higher concentration (200 mg/L), and it was detected even less than the control with 25%. Stem lengths were unaffected by concentration variation (P ≥ 0.05). The average root length for all administrations was ± 26.2 mm. The concentration-dependent variability of *GVI* was due to the difference in germination percentage, as did the *GI*. The maximum increase rate of GVI was determined with a 12% at 100 mg/L, due to the germination percentage (21%) showing the highest value at 100 mg/L concentration, compared to control. In this case, it can be thought that the proper concentration of Fe_3_O_4_-NPs improves the germination process by increasing the seed water intake, especially with the increase in permeability in the seed coat, during the initial stages of seed germination. Fe_3_O_4_-NPs photocatalyst activity^33^, and rising concentrations of Fe_3_O_4_-NPs positively affected germinating basil seeds. Because photocatalysts have the ability to convert solar energy into chemical energy rapidly, they form strong oxidizing species such as oxide, peroxide and hydroxyl radicals on the surface with the effect of absorbed sun-ray, enabling the conversion of harmful organic molecules into harmless species such as water and carbon dioxide^34^ and so this accelerates, the photoreaction. On the other hand, the catalytic effect of NPs (through accelerating photoreactions) due to surface/volume relationship and high surface charges explains the best results of all germination data examined at 100 mg/L Fe_3_O_4_-NPs concentration^35^. A similar state was found in seeds where seed fresh weights (*FW*) were measured at the end of germination. Seed weights changed with increasing concentration (Fig. 3.).The maximum seed weight was 0.205 g at 100 mg/L concentration of Fe_3_O_4_-NP, although this value decreased at lower and higher concentrations (P ≤ 0.05). Fe_3_O_4_-NPs applied to barley seeds significantly increased plant biomass at a similar concentration (100 mg/L)-compared to other concentrations- the maximum increase was found in root biomass.

The dry weights (*DW*) of seeds have not been affected by the concentration increase (*P* ≥ 0.05). The seed water content (*SWC*) was come up by differences in fresh weights that were a result of the variation in NP concentration. Accordingly, the highest value of *SWC* was determined with 87% at 100 mg/L concentration of Fe_3_O_4_-NPs. The tendency to decrease of *SWC* at 200 mg/L (76%) is due to the blockage in water and nutrient uptake by the high NP concentration causing occlusion in the apoplast. Since the penetration of the appropriate concentration of Fe_3_O_4_-NPs into the seed coat accelerates root emergence by preventing, abnormal cell formation in mitotic cell division in root tip cells that allow the uptake of water, inorganic ions, and other nutrients. It was determined that the high surface reactivity of NPs showed a significant increase in germination percentage by increasing the hydro-mineral movement in the roots, expanding or increasing the number of root pores that promote nutrient uptake^36^.

## Conclusion

**Figure 4:**
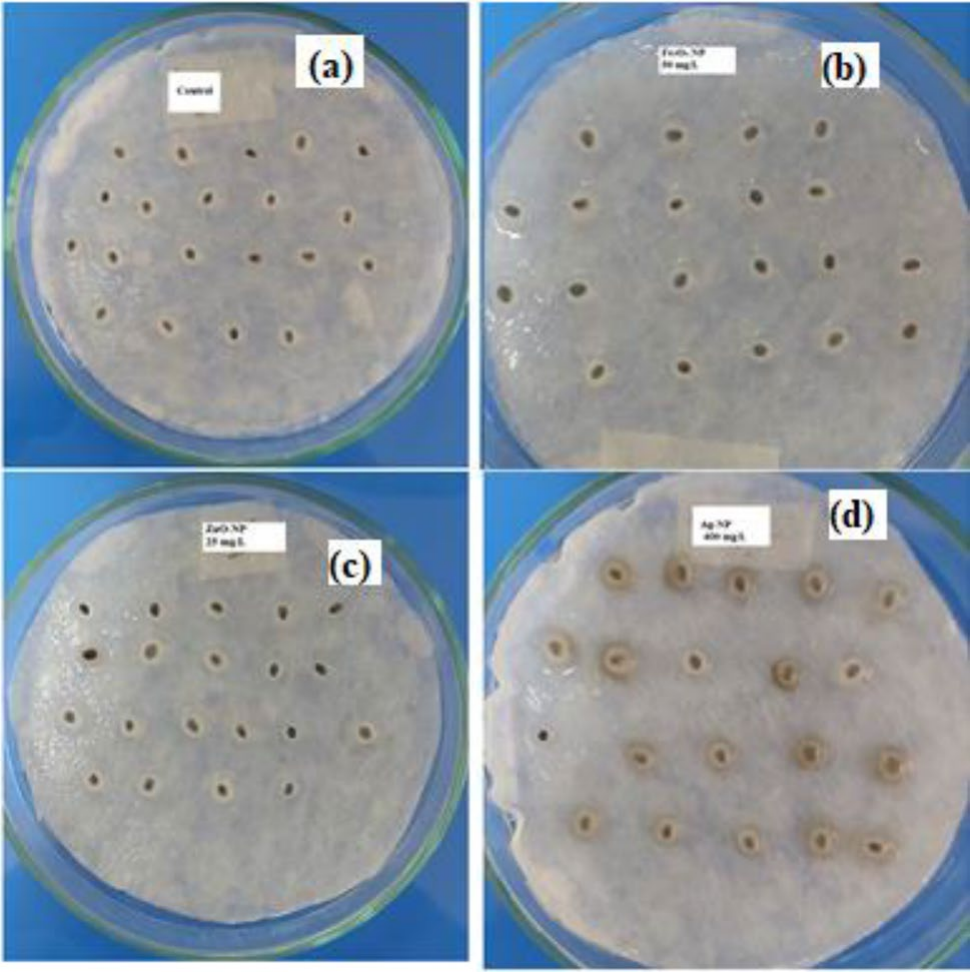
The water holding capacity of basil seeds germinated at different concentrations of NPs on the third day of germination: a. control; b. Fe_3_O_4_-NP (50 mg/L); c. ZnO-NP (25 mg/L); d. Ag-NP (400 mg/L).

The intake and regulate of water to the seed, which is defined as the beginning of germination, is effective in sustaining various physiological events in all vital processes of the plant. Since *GI* and *GVI* data, which are indicators of healthy and quality seed germination, are a guarantee of good growth, these data are important data in determining the NP and its concentration, which are effective in the germination of basil seeds. On the other hand, the mostly spherical shape of the biosynthesized NPs in this study significantly stimulated the germination ability of basil seeds, as their activity was higher than the rod-shaped NPs. At the same time, the sizes of the biosynthesized NPs also varied. Ag-NPs were detected at 15–28 nm, ZnO-NPs at 50–100 nm, and Fe_3_O_4_-NPs at 21–27 nm. The presence of numerous and various combinations of phytochemicals acting as reducing agents in the plant extract may be the cause of the size variation within each NP. The fact that Ag-NPs has the highest root length of 19.4 mm at 200 mg/L, compared to all NP applications, may be due to the fact that AgNO_3_, which is an anti-ethylene agent, causes changes in phytohormone levels by inhibiting ethylene receptors. Since changes in seed imbibition rate trigger transcriptomic changes such as lipid metabolism, auxin homeostasis, and upregulation of gibberellin related to germination, a linear relationship can be established between germination and seed water content. While *SWC* value was 76% in control, 90% at ZnO-NP 25 mg/L, and 86% at Fe_3_O_4_-NPs 50 mg/L, *SWC* was determined to be maximum with 120% in Ag-NPs 400 mg/L. Since Ag-NPs form the seeds with maximum water content when administered to basil seeds at 400 mg/L concentration, it may be deduced that this NP (Ag-NP) is the NP concentration that stimulates the ability to absorb water of seeds the most in comparison to the others (Fig. 4.). This can also be explained by the presence of a water layer (hydrogel) of varying thickness around the basil seeds with Ag-NP concentrations. Thanks to this layer, which disappears after the first 3rd day of germination, the SWC results are similar to the water capacity of the petri dishes and the thickness of the water bands around the seeds during the germination trials, and Ag-NPs can provide the optimum use of the water required during the germination period, with the water-retaining feature. Ag-NP concentrations that are appropriate for germination and seedling growth increase water uptake into seeds and storage reserve metabolism, such as starch, by permitting membrane permeability to allow water and small molecules to move through rapidly. On the other hand, the proportional relationship between root length and seed water content indicates that the best germination is at 200 mg/L Ag-NPs. At the same time, the fact that the germination index was determined at the highest value at this concentration compared to other NP applications (139.15%) indicates that Ag-NPs is the application that best achieves nutrient transfer from the cotyledons to the embryonic axis. In this way, the germination ability reaches the maximum level. Since they don't include any hazardous substances and have effects that improve soil quality and soil content, the using optimum amounts of Ag-NPs, ZnO-NPs and Fe_3_O_4_-NPs produced by phytonanosynthesis suggest that they may be products that offer unique opportunities to grow sustainable crops.

## Data Availability

The datasets used and/or analysed during the current study available from the corresponding author on reasonable request.
